# A new species of *Sarcophaga* (*Pandelleisca*) (Diptera, Sarcophagidae) from Turkey

**DOI:** 10.3897/zookeys.937.50759

**Published:** 2020-06-01

**Authors:** Gamze Pekbey

**Affiliations:** 1 Department of Plant Protection, Faculty of Agriculture, Yozgat Bozok University, Yozgat-Turkey Yozgat Bozok University Yozgat Turkey

**Keywords:** Anatolia, flesh fly, identification, Mediterranean region, Mersin, Middle East

## Abstract

A new species, Sarcophaga (Pandelleisca) mersinensis**sp. nov.** is described from the Mediterranean region of Turkey. The male terminalia are documented with line drawings, photographs and scanning electron microscope images. The species is compared with the two most similar species, Sarcophaga (Pandelleisca) baudeti (Lehrer) and Sarcophaga (Pandelleisca) theodori (Lehrer), both known from Israel. A key is provided to the western Palaearctic species of *Pandelleisca* Rohdendorf.

## Introduction

The subgenus Pandelleisca Rohdendorf, 1937 (of *Sarcophaga* Meigen, 1826) contains 24 species of flesh flies, mainly distributed in the Oriental region (Verves 1986; Sugiyama et al. 1990; Pape 1996; Lehrer 1998, 2008; Kurahashi and Leh 2007; Piwczyński et al. 2014). Most species are Oriental or eastern Palaearctic, and only three species have so far been recorded from the western Palaearctic: S. (P.) baudeti (Lehrer, 1998) and S. (P.) theodori (Lehrer, 1998), both known only from Israel, and S. (P.) similis Meade, 1876, which is widely distributed in both the Palaearctic and the Oriental regions (Pape 1996; Lehrer 1998). Sarcophaga (P.) similis is the only representative of the subgenus recorded in Turkey so far (Kara and Pape 2002; Verves et al. 2018).

The assignment of *Pandelleisca* at either the generic or subgeneric level differs among authors. The nominal taxon was erected by Rohdendorf (1937) as a subgenus in his broad concept of the genus *Parasarcophaga* Johnston & Tiegs, 1921, with the designation of *S. similis* as type species. Subsequently, Pape (1996) employed a broad generic concept and placed *Pandelleisca* within the genus *Sarcophaga*.

The general morphological outline of the phallus within species of *Pandelleisca* appears remarkably similar to what is found in the subgenus Liosarcophaga; however, *Pandelleisca* has been separated from *Liosarcophaga* essentially due to having a massive, long and well-sclerotized paraphallus with a broader and larger median process of juxta, one or two pairs of curved lateral juxtal arms, two-paired and spiky vesical lobes, thin and long styli, and absence of the marginal bristles on the genital tergite (Rohdendorf 1937; Tumrasvin and Kano 1979; Povolný 1987; Povolný and Verves 1997; Peris et al. 1999).

This paper describes a new species of Sarcophaga (Pandelleisca) from the Mediterranean region of Turkey, providing photographs, scanning electron microscope (SEM) images, and line drawings of the male terminalia, and a key to the western Palaearctic species.

## Materials and methods

The material was collected during the years 2013–2017 in Mezitli and Erdemli districts of Mersin Province of Turkey using insect sweep nets. The specimens were killed in ethyl acetate vapour, pinned shortly afterwards when they were still fresh and air-dried.

Males were relaxed in a humidifier, and the terminalia of each specimen were detached from the abdomen using forceps and fine insect pins. The dissected terminalia of the holotype were subjected to 10% KOH for 12 hours, rinsed with distilled water and placed into glycerine for further examinations under a Leica S8APO stereomicroscope.

The air-dried genitalia of the paratype were prepared for SEM by fixing on an aluminium stub with carbon double-stick tape. The gold-coated specimens were examined and imaged in a FEI Quanta 450 FEG scanning electron microscope at BILTEM (Science and Technology Application and Research Centre of Yozgat Bozok University) using high vacuum.

Light microscope photographs were taken with a Leica DFC 450 camera integrated on a Leica M125 stereomicroscope and stacked in Helicon Focus Pro (version 7.6.1). Line drawings of terminal structures were produced with CorelDraw Graphics Suite 2019.

The terminalia of the holotype of the new species are stored in a micro-vial with glycerine and the dissected parts of the terminalia of the paratype were glued to a piece of card and both are pinned together beneath the source specimens. All the samples are deposited in the Entomology Collection of the Department of Plant Protection, Faculty of Agriculture, Bozok University, Yozgat, Turkey.

For identification, the following works were consulted: Böttcher (1912), Senior-White (1924), Rohdendorf (1937), Tumrasvin and Kano (1979), Nandi (1982), Sugiyama et al. (1988, 1990), Kurahashi and Leh (2007), Lehrer (1998, 2008), Kurahashi and Chaiwong (2013). The nomenclature and classification follow Pape (1996). The terminology of external morphology and terminalia follow Richet et al. (2011) except for vesical lobes where the terms “superior vesical lobes” and “inferior vesical lobes” are used as adopted by Lehrer (1998) to provide a detailed description of these structures. The comparisons of *S﻿.* (*P.*) *baudeti* (Lehrer, 1998) and *S.* (*P.*) *theodori* (Lehrer, 1998) with the other western Palaearctic species were based on the original descriptions of Lehrer (1998).

Data from labels of the type specimens are quoted verbatim: commas are used to separate the lines on the same label, labels are separated by a double forward slash, and any remarks are given in square brackets.

## Results

### Sarcophaga (Pandelleisca) mersinensis sp. nov.

Taxon classificationAnimaliaDipteraSarcophagidae

1736C6B6-E816-5E24-87F6-D9AD463A4187

http://zoobank.org/31EF981D-0E7D-459D-98F7-F4D6815DABC3

Figures 1 , 2 , 3

#### Type material.

***Holotype***: ♂, TR// Mersin province [southern Turkey], Mezitli district, 1.2 km NE Kuzucu village, 608 m, 36°50'32"N, 34°25'24"E, 07.VII.2017, Leg. G. Bakır [printed on white paper] // Holotype ♂ Sarcophaga (Pandelleisca) mersinensis, Det. Pekbey, 2020 [printed on red paper]. ***Paratype***: ♂, TR// Mersin province [southern Turkey], Erdemli district, Kösbucağı village, 542 m, 36°40'58"N, 34°14'37"E, 11.VII.2013, Leg. C. Metin [printed on white paper]. Sarcophaga (Pandelleisca) mersinensis, Det. Pekbey, 2020 [printed on red paper].

#### Differential diagnosis.

**Figure 1.**
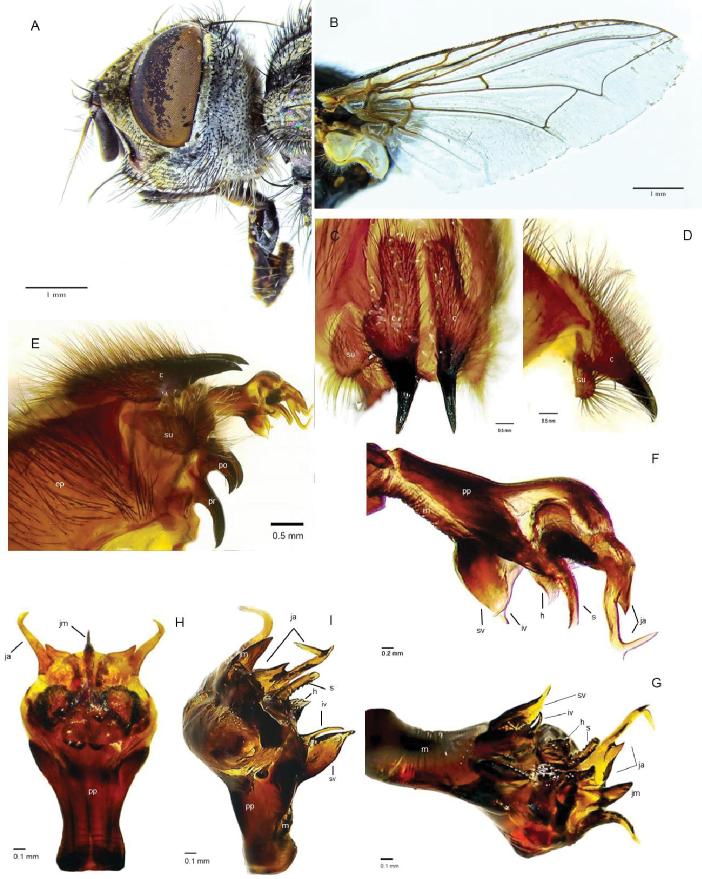
Sarcophaga (Pandelleisca) mersinensis sp. nov., male holotype **A** head, left lateral view **B** wing, ventral view **C** cerci and surstyli, dorsal view **D** cerci and surstyli, right lateral view **E** terminalia, right lateral view in glycerine **F** distiphallus, right lateral view in glycerine **G** distiphallus, right lateroventral view **H** distiphallus, dorsal view flipped vertically **I** distiphallus, right laterodorsal view flipped vertically. Abbreviations: c, cerci; ep, epandrium; h, harpes; iv, inferior vesical lobe; ja, lateral juxtal arms; jm, medial part of juxta; m; membrane; po, postgonite; pp, paraphallus; pr, pregonite; s, styli; su, surstyli; sv, superior vesical lobe.

**Figure 2.**
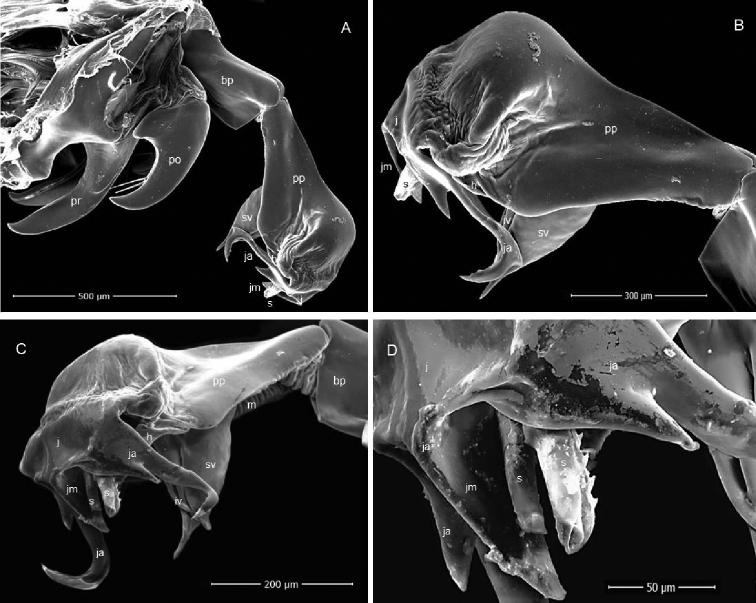
SEM microphotography of Sarcophaga (Pandelleisca) mersinensis sp. nov. terminalia, male paratype **A** habitus of phallus and gonites, right lateral view **B** distiphallus, right lateral view flipped horizontally **C** distiphallus frontolateral view **D** apical part of distiphallus. Abbreviations: h, harpes; iv, inferior vesical lobe; ja, lateral juxtal arms; jm, medial part of juxta; m; membrane; po, postgonite; pp, paraphallus; pr, pregonite; s, styli; sv, superior vesical lobe.

**Figure 3.**
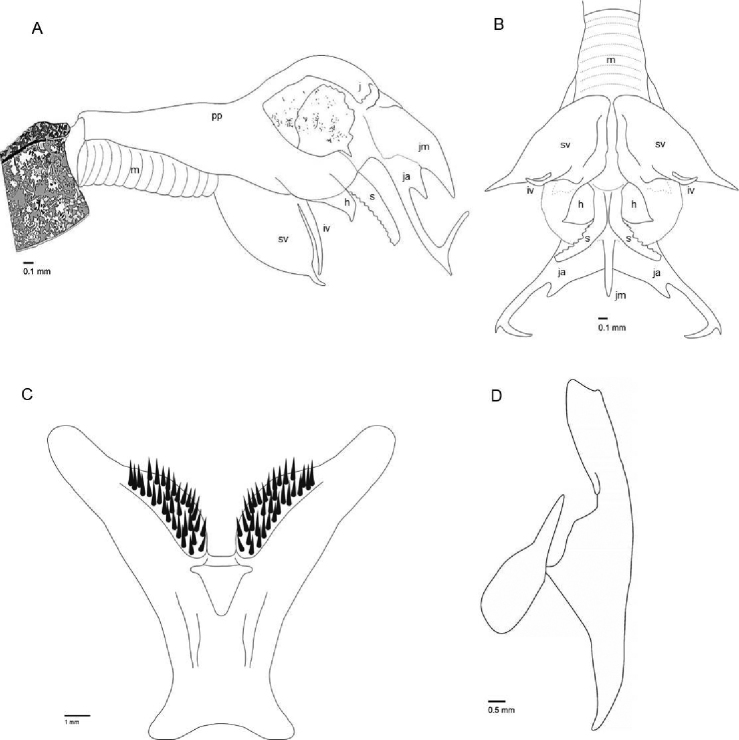
Sarcophaga (Pandelleisca) mersinensis sp. nov., male, holotype **A** distiphallus, right lateral view in macerated terminalia **B** distiphallus, ventral view in macerated terminalia **C** ST5, frontal view **D** cerci and surstyli, right lateral view. Abbreviations: h, harpes; iv, inferior vesical lobe; ja, lateral juxtal arms; jm, medial part of juxta; m; membrane; pp, paraphallus; s, styli; ST5, sternite 5; sv, superior vesical lobe.

Sarcophaga (Pandelleisca) mersinensis sp. nov. is similar to the East Mediterranean species S. (P.) theodori (Lehrer, 1998) and *S.* (*P.*) *baudeti* (Lehrer, 1998). It is distinguished from *S.*﻿ (*P.*﻿) *baudeti* by having a brown epandrium (Fig. 1E), and it differs from *S.*﻿ (*P.*) *theodori* by the following features of the male terminalia: in *S.*﻿ (*P.*﻿) *mersinensis* the harpes are subtriangular in lateral view, poorly sclerotized and very small (Figs 1F, G, I; 3A, B) that they can be easily overlooked due to shrinkage and overlapping by the long and broad ventral projections of the phallus in dry genitalia (Fig. 2A, B). In macerated specimens, the harpes lie anteroventrally from the base of the lateral styli but never reach beyond these (Fig. 1F, G, I). The superior vesical lobe extends to a long and pointed end. The lateral styli are flattened and serrated along the entire ventral margin. Surstyli are narrow and rounded distally (Figs 1F–I; 2A–D; 3A, B). Cercal prongs are blackish (Fig. 1C, D). Postgonites show a pair of bristles just distal to the middle on ventral surface (Figs 1E, 2A).

#### Description.

**Male.** Body length 10.7–11.2 mm (without terminal extension).

***Head.*** Black with golden microtomentum and the eye 0.38 times as wide as head in dorsal view. Inner vertical seta long and strong, outer vertical and proclinate orbital seta indistinct. Reclinate orbital seta well developed. Eye bare. Postocular seta black, arranged in two rows on each side of occiput. Frons apically protruding and at its narrowest point 0.68 times as wide as an eye in dorsal view. Frontal vitta black, slightly widening to antennal insertion, 0.48 times as wide as frons. Frontal bristles 11 or12 pairs, not descending below of the midline of pedicel. Parafacial plate black with golden microtomentum, with a row of fine and black setulae in lower half near eye margin. Parafacial at its narrowest point 0.42 times as wide as an eye at maximum eye width in lateral view. Gena black with golden-silvery microtomentum, anterior half covered with black seta, post genal seta pale. Gena in profile 0.36 times as high as the height of an eye. Genal dilation distinct, brownish black. Vibrissa well developed. Facial ridge with a few decumbent setulae above vibrissa. Antenna brownish black, pedicel with a reddish-brown tinge on the distal part. Postpedicel 2.76 times longer than pedicel. Arista light brown, 2/3 plumose, slightly thickened on basal part. Prementum and palpus dark brown, 2.2 times longer than wide (Fig. 1A).

***Thorax.*** Black with silver microtomentum with three black longitudinal stripes. Anterior stigma brown, posterior one bright yellow. Propleuron bare. Prosternum and postalar wall setulose. Acrostichals 0+1, dorsocentrals 4+4, presutural and first two postsuturals short and reduced, intra-alars 1+2, presutural 1, supra-alars 3–4, humerals 3, posthumerals 2, notopleurals 4 (2 primary + 2 subprimary), katepisternals 2 + 1; scutellum with two pairs of subapical setae, one pair of basal and one pair of discal setae.

***Legs*** black. Fore tibia with three anterodorsal and one posteroventral seta. Mid femur with scarce and short ctenidium. Mid tibia with two or three anterodorsal, one anteroventral, and three or four posteroventral setae. Hind tibia with a row of hair-like setae on posteroventral and ventral surface, with two strong anterodorsal setae, 1 anteroventral and one posterodorsal.

***Wing*.** Hyaline. Epaulet black. Basicosta bright yellow. Costal spine absent. Vein R_1_ bare. Vein R_4+5_ dorsally with short and black setulae at base. Distal part of M curved at a right angle. Second costal section 1.44 times as long as fourth costal section. Cell r_4+5_ open at wing margin. Haltere brown. Lower calypter yellowish white (Fig. 1B).

***Abdomen***. Black with silvery microtomentum with small checkerboard patterns changing with the incidence of light. Syntergite I+II and tergite III without median marginals. Tergite IV with a pair of median marginals. Tergite V with a complete row of marginal setae.

***Terminalia.*** Sternite 5 V-shaped, elongated and slightly indented medially at base; arms of sternite 5 flattened with a median expansion with a bunch of short and stout setulae proximally along the inner margin of each arm (Fig. 3C). Syntergosternite 7+8 brownish and subrectangular, without marginal setae. Epandrium brown with irregular fine and long setulae (Fig. 1E). Base and body of cerci brown and setose, dilated in the midline posteriorly (Fig. 1C). Cercal prongs dark, bare on ca 2/3 of dorsal surface, nearly straight with the exception of a median protuberance ventrally, descending to the middle, slightly curved and terminated with a more or less pointed apex in lateral view (Fig. 1C, D). Surstyli brown, elongated, rounded distally, and covered with long black setae (Figs 1C, D; 3D). Gonites dark brown; pregonite long and compressed with a slight convex curve of the ventral surface and a pointed tip; postgonite short and robust, hook shaped with two median bristles ventrally (Figs 1E; 2A). Phallus brown; basiphallus nearly 1/2 length of phallus and with an articulated connection to paraphallus. Paraphallus and juxtal plate well sclerotized; median part of juxta long and blade-like, bent in a right angle apico-ventrally. Lateral arms of juxta narrow, bipartite and nearly 0.50 times as long as distiphallus; basal projection of juxtal arm short and spur-shaped, distal projection long and sharply upturned at the end with a right angle and asymmetrically forked at the tip (i.e., at the bend). Lateral styli long and slender, extended beyond 2/3 the length of the lateral juxtal arms and serrated throughout the ventral surface (Figs 1F–I; 2A–D; 3A, B). Harpes membranous, small, and subtriangular in lateral view, not reaching beyond half-way along the lateral styli and only visible in macerated terminalia (Figs 1F–I; 3A, B). Vesica bilobed. The superior lobe leaf-like, compressed, greatly enlarged, inferior lobe short and narrow. Each lobe sharply pointed at the tip (Figs 1F–I; 2A–D; 3A, B).

**Female.** Unknown

#### Biology.

Unknown

#### Distribution.

Palaearctic – Turkey (Mediterranean region of Anatolia, Mersin).

#### Etymology.

The species epithet is derived from Mersin Province situated in the Mediterranean region of Turkey, where the type series was collected.

## Key to the western Palaearctic species of the male Sarcophaga (*Pandelleisca*) (Rohdendorf, 1937)

**Table:** 

1	Median part of juxta with a strong tooth on the ventral surface; lateral juxtal arms not expanding basally and narrow at the tip; each vesical lobes poorly sclerotized, slender and nearly filamentous in lateral view; sternite 5 roughly Y-shaped	**S. (P.) similis**
–	Median part of juxta without a strong tooth on the ventral surface; lateral juxtal arms expanding basally and bi-paired at half-way; vesical lobes more or less sclerotized; superior vesical lobes leaf-like in lateral view; sternite 5 V-shaped	**2**
2	Lateral juxtal arms narrow at the tip with an axe-shaped basal expansion; cerci medium sized, relatively widening at midline, cercal prongs flat and slightly bending outwards at apex; surstyli narrow and long; epandrium black	**S. (P.) baudeti**
–	Lateral juxtal arms slightly forked at the tip with a spiky and pointed tip basal expansion; cerci relatively short, dilated in midline, cercal prongs slightly curved and terminating with a more or less pointed apex; surstyli more or less quadrangular, with round corners; epandrium brown	**3**
3	Harpes triangular, narrow, and reaching beyond lateral styli. Lateral styli with recurving teeth ventrally on the distal 1/3 at most	**S. (P.) theodori**
–	Harpes subtriangular, small and never reaching beyond lateral styli. Recurving teeth extending along the entire ventral surface of lateral styli (Figs 1F, G, I; 2A–D; 3A, B)	**S. (P.) mersinensis sp. nov.**

## Discussion

Thirteen species of *Pandelleisca* are recorded only from the Oriental region, and the six Palaearctic species are restricted to the far eastern territories including Palaearctic China, Russia, North Korea, South Korea, and Japan (Rohdendorf 1937; Verves 1986; Sugiyama et al. 1990; Pape 1996; Kurahashi and Leh 2007; Kurahashi and Tan 2009). *Sarcophaga* (*P.*) *similis* is widely distributed throughout the Palaearctic and Oriental regions and is found mostly in mesophytic forest habitats (Povolný and Verves 1997). The species has also been recorded in Turkey from the coastal provinces such as Aydın and Muğla in the Aegean part, and Trabzon of the Black Sea area (Kara and Pape 2002; Verves et al. 2018). The newly described species *S.* (*P.*) *mersinensis* was collected from the Mediterranean coastal region as are the two Israeli species, *S.* (*P.*) *theodori* (Lehrer 1998) and *S.* (*P.*) *baudeti* (Lehrer, 1998).

*Sarcophaga* (*P.*) *theodori* is the species most similar to *S.* (*P.*) *mersinensis* sp. nov. with regard to morphological structures of the phallus. In both species, the distiphallus expands abruptly apicolaterally in dorsal view and has elongated ventral appendages. The median process of juxta is broad, flattened, and spur-like in lateral view in both species, and bends anteroventrally with a wide angle towards the lateral juxtal arms. These arms are paired, widened basally, and slightly bifurcated at the tip. The superior vesical lobes are leaf-like, and the inferior ones are relatively thin and spiky. The lateral styli are broad and tubular in both species.

As stated by some authors, the vast majority of *Pandelleisca* species have a shiny black epandrium (Rohdendorf 1937; Verves 1986; Sugiyama et al. 1990; Lehrer 1998; Peris et al. 1999; Kurahashi and Leh 2007; Lehrer 2008), but in a few species such as *S.*﻿ (*P.*) *ballardi* (Senior-White, 1924), *S.* (*P.*﻿) *brachiata* (Sugiyama, 1990), *S.* (*P.*﻿) *quinqueramosa* (Sugyiama, 1990), and *S.* (*P.*) *theodori* (Lehrer, 1998) the colouration of the epandrium is brown as in *S.* (*P.*) *mersinensis* sp. nov.

The taxonomic limits between *Pandelleisca* and *Liosarcophaga* are still unsettled. Some molecular studies have shown that *Liosarcophaga* and *Pandelleisca* are not monophyletic in their current circumscriptions, and that the subgenus Pandelleisca as proposed by Pape (1996) is paraphyletic or even polyphyletic (Song et al. 2008; Ming et al. 2014; Piwczyński et al. 2014). On the other hand, Piwczyński et al. (2014) provided evidence, that five species formerly assigned to different genera from the Oriental and eastern Palaearctic regions are better grouped within the subgenus Pandelleisca. The phylogenetic placement of *S.* (*P.*) *similis* in the study of Piwczyński et al. (2014) was particularly striking, because it was recovered as the sister taxon of Sarcophaga (Rosellea) aratrix Pandellé rather than grouping with all other species here considered under *Pandelleisca* (Piwczyński et al. 2014). This must, however, be under strong suspicion of being an example of one of the many misidentifications that are well-known within the genus *Sarcophaga*.

In conclusion, although all *Pandelleisca* species appear to be recorded from the coastal geographic regions in Turkey, it is thought that future more comprehensive faunistic surveys will reveal the true distribution of this subgenus.

## Supplementary Material

XML Treatment for Sarcophaga (Pandelleisca) mersinensis
